# Anti-Monopoly

**Published:** 1881-11

**Authors:** 


### ANTI-MONOPOLY.

NO one believes that the relations existing between the public and the railroad companies are entirely satisfactory; but to find the remedy, "that's the question." It seems to me that a frank, truthful statement of the grievances complained of would accomplish the ends sought for much sooner than the blustering and defiant plan that seems to have been adopted in some instances. For example, what could have been more absurd than the resolution passed by the "Anti-Monopoly League," in Jersey City, a few weeks ago, to the effect that the Pennsylvania Railroad Company should be prohibited from running their locomotives into the city. I am not certain but that the resolution came indirectly from the shrewd managers of the railroad company, as nothing so soon destroys a movement of this kind as to make it ridiculous. The effect of this resolution, if it could ever be carried out, would not only be to destroy the business of the railroad company, but to impair the value of property in Jersey City and along the line of the road, and to depopulate that portion of the country.

The company could hardly afford to break their long trains at Marion and jog to the ferry with four horses attached to each car; and no business man in a hurry to get to New York would enjoy more than one such trip.

---

Salt for Farm Stock.—Professor James E. Johnson, of Scotland, says that half the saline matter of the blood consists of common salt, and as this is partly dissolved every day through the skin and kidneys, the necessity of continued supplies of it to the healthy body is sufficiently obvious. The bile also contains soda (one of the ingredients of salt) as a special and indispensable constituent, and so do all the cartilages of the body. Stint the supply of salt, and neither will the bile be able properly to assist digestion, nor the cartilages to be built up again as fast as they naturally waste. It is better to place salt where stock can have free access to it, than to give it occasionally, in small quantities. They will help themselves to what they need, if allowed to do so at pleasure; otherwise, when they become salt-hungry, they may take more than is wholesome.

---

A great many men are like a rocking-horse. They are always on the go, but never get ahead.

